# Relationship between resilience, optimism, and burnout in Pan-American athletes

**DOI:** 10.3389/fpsyg.2022.1048033

**Published:** 2022-11-14

**Authors:** Veronica Tutte-Vallarino, Estefanía Malán-Ernst, Mario Reyes-Bossio, Ana Peinado-Portero, Julio Imbernón de Álvaro, Francisco José Ortín Montero, Enrique J. Garcés de Los Fayos Ruiz

**Affiliations:** ^1^Departamento de Bienestar y Salud, Universidad Católica del Uruguay, Montevideo, Uruguay; ^2^Facultad de Psicología, Universidad Peruana de Ciencias Aplicadas, Lima, Peru; ^3^Facultad de Psicología, University of Murcia, Murcia, Spain

**Keywords:** resilience, optimism, burnout, athletes, high performance

## Abstract

**Aim:**

A series of knowledge has been developed on burnout syndrome in the sports context that has allowed to generate a solid theoretical structure that requires new contributions to delve into those aspects that have been less addressed, as is the case of optimism and resilience when it comes to linking them to the syndrome in top-performance athletes. For this purpose, the Burnout Inventory for Athletes (IBD-r), the Resilience Scale adapted to Spanish, and the Life Orientation Scale-Revised (LOT-R) for optimism were used.

**Methods:**

From this perspective, the study was approached with 121 Uruguayan athletes who participated in the last Pan-American Games, assuming practically all the subjects who have this level of sport (142) considering gender and type of sport.

**Results:**

The results indicate that 67% of the sample presents burnout symptoms for the emotional exhaustion dimension, and moderate resilience and optimism. Although there are no significant differences attributable to gender or type of sport, this contribution allows us to continue focusing on future work and further analysis. The type of sport presented statistically significant differences in relation to the personal competence dimension and the type of sport.

**Conclusion:**

Resilience and optimism obtained a clear influence on the occurrence of burnout, in a statistically negative sense, showing themselves as interesting prevention strategies for future lines of research, where it is essential to design interventions that teach emotional skills to manage adversity and prevent burnout.

## Introduction

At present, we can state that the social conception of sport has not changed, being understood as one of the activities with the greatest impact on the world population, either because it is an activity carried out by the population or because it is followed, passively, by millions of spectators. In any case, the generation of pressure on the protagonist (the athlete) continues to increase, and the athlete perceives it as a sign that, in many cases, can become a problem: the first time that excess pressure, related to burnout, became an objectifiable concern in the field of elite sport was during the development of the Barcelona Olympic Games or, at least, it was made explicit in a clear way, when May (1992) pointed out that burnout had been one of the psychological problems that, as a sport psychologist, he had to deal with.

A construct that was hardly known in the field of research was becoming evident, although the syndrome had appeared in the scientific literature a few years earlier, when Freudenberger (1974), in the context of clinical psychology, described a person presenting a previously unknown psychopathological picture. Before May (1992), the first works on burnout in sport had appeared, by pioneering authors such as Flippin (1981) and Feigley (1984), who already pointed out the importance that the suffering of this syndrome could have for athletes who, because of persistent and lasting stress, could show a variety of problems.

From these pioneering works, others followed showing the wide involvement in both health and sporting activity of athletes (Goodger et al., 2007), since the prevalence found by the different authors was beginning to be striking. This is the case of De Francisco et al. (2014) and Olivares et al. (2018) who found about 4% of athletes with burnout. Gustafsson (2007) estimated the prevalence of this problem in athletes to be between 1 and 9%. And other subsequent studies placed it at least 10% (Gustafsson, 2007; Hodge et al., 2008; Dubuc-Charbonneau et al., 2014; Ziemainz et al., 2015).

We would therefore be facing an important problem for athletes and for the professionals who work with them (coaches, physical trainers, sports psychologists, etc.), as well as for their closest family members, as they are the people of reference when the problematic situation is present. For all these reasons, research on the subject has been progressively increasing, especially in terms of empirical work aimed at obtaining more information about the syndrome (Goodger et al., 2007; Marín et al., 2013; Lundkvist et al., 2018; Reche et al., 2018a; Wagstaff et al., 2018).

In accordance with the above, the work we present here would be framed in the scientific discourse developed around burnout in athletes, since our aim is to know the relationship between two essential psychological variables in the performance of these athletes, whether or not they present burnout. We refer to resilience and optimism, two aspects that must be properly tuned in the person who practices competitive sport, especially if it is high-performance sport, as is the case here.

It is assumed that the demands of this elite sport require athletes with at least two fundamental characteristics, among others, as highlighted in the work of García-Jarillo et al. (2020): resilience and optimism. Thus, one of the variables that are being most studied in the field of sports is resilience (Bretón et al., 2016) because it allows the athlete an attitude of overcoming adversity (Ortín et al., 2013) and an influence on the improvement of their athletic performance (Romero, 2015).

Resilience is a multidimensional construct that is related to balanced personality profiles (Friborg et al., 2005), and to people able to flexibly change their affective and physiological responses to adapt to the demands of the environment (Waugh et al., 2011). In the sport context, we find two theoretical models that explain the importance of sport-related resilience. One of them is that of Fletcher and Sarkar (2012), who state that athletes with resilient characteristics evaluate stressful situations as a motivating challenge and not as a threat. The second is that of Galli and Vealey (2008), who indicate that what is important in the resilience process is to be able to perceive that they have achieved positive results because of having faced adversity.

García-Secades et al. (2014) make a classification of the psychological variables that affect resilience dividing between risk factors (stress, negative affect, anxiety, depression, and post-traumatic stress) and protective factors (self-efficacy, self-esteem, social support, and optimism). In addition, psychosocial factors contributing to resilience include optimism, autonomy, self-confidence, coping strategies, feelings of self-efficacy, physical exercise, humor, and prosocial behavior, among others (Wu et al., 2013). Among them, one of the most related factors that contributes to the development of resilience is optimism (Wu et al., 2013; Reche et al., 2018b), a personality construct that has been shown to play a very important role in sports performance (Wilson et al., 2002; Gordon, 2008).

Optimism refers to the stable belief that positive events will occur and the ability to make the best of the experiences lived, always from a realistic vision and not from denial or excessive optimism that flees from reality (Gómez-Díaz, 2016). In the sports context, optimism has been studied as a personality trait in athletes (Gould et al., 2002), and as a factor that influences in relation to stress (Albinson and Petrie, 2003). The scientific literature states that the optimistic athlete has better prognoses of success and situational stress control than the pessimist (Seligman et al., 1990; Ortín et al., 2011).

Recent studies significantly associate optimism and resilience (Yu and Zhang, 2007; Parkes and Mallet, 2011; Souri and Hasanirad, 2011; Freche, 2013; Tutte and Reche, 2016; Cortez-Saldarriga et al., 2022; Lacárcel et al., 2022). In addition, it seems that the most optimistic athletes (who tend to be also the most resilient) present less anxiety and greater self-confidence, in addition to better performance (Martin-Krumm et al., 2003). In fact, Wu et al. (2021) highlight the importance of resilience and anxiety in understanding how stressors are related to syndromes such as burnout. In this sense, those athletes who were less resilient and more prone to burnout were more likely to abandon sports practice (Sorkkila et al., 2019).

It is at this context where the work we present fits in, seeking through our main objective to describe the incidence of burnout in Uruguayan athletes of the highest competitive level, and in turn, to relate resilience, optimism, and burnout syndrome in line with the recent work of Olivares (2021), in which the author highlighted the need to analyze different psychological variables that could provide reasonable alternatives to the proposed explanation in the origin and development of the syndrome.

Likewise, the importance of establishing the predictive power that resilience and optimism could have on the possibility of developing burnout or not in high-performance athletes.

## Materials and methods

### Participants

A total of 121 high-performance athletes from Uruguay who participated in the last Pan-American Games in Lima were evaluated. Their ages ranged between 14 and 52 years (*M* = 24.01; SD = 6.2), corresponding to 28 different sports, 72 participants (59.5%) competed in individual sports and 49 (40.5%) in team sports. In relation to gender, 53 athletes were female (43.8%) and 68 were male (56.2%).

The type of sampling used is non-probabilistic by convenience, since there were inclusion criteria (participating in the Olympic cycle, being qualified or in the process of qualifying for the Pan-American Games, or being a federated athlete). It is important to clarify that this sample represents practically the total number of athletes who participated in these games, a total of 142. In other words, the sample evaluated represents 85% of the total population that participated in these Games. Table 1 shows the aspects mentioned above.

**Table 1:** Contingency table of players according to gender and level of competition.

		Individual	Collective	Total
Gender	Feminine	27 (37.5%)	26 (53.1%)	121
	Male	45 (62.5)	23 (46.9%)	121
Total		121	121	

### Evaluation instruments

To obtain a complete evaluation of the athletes, an *ad hoc* form was developed that included informed consent, sociodemographic data of the athletes, gender, and type of sport (individual or collective). Three measurement instruments were used to assess the variables optimism, resilience, and burnout. The Spanish version of the Life Orientation Scale-Revised (LOT-R) by Otero et al. (1998) was used to assess optimism. This scale evaluates individuals’ expectations of positive or negative outcomes about the future. It is composed of ten items, with a Likert-type response format, from 0 (totally disagree) to 4 (practically always agree), where 4 of the items are control items, 3 are about positive expectations, and 3 are about negative expectations. The scale has an internal consistency of 0.78. This instrument allows, first of all, to analyze each disposition independently (optimism and pessimism) by adding the items of each subscale, and subtracting the values obtained in optimism, and those of pessimism. In this way, we obtain the optimistic tendency of a subject if he/she has obtained positive values (between 0 and 12 points) and negative tendency if he/she has obtained negative values (between 0 and −12). Scores from −12 to 2 are considered low optimism, from 3 to 5 medium optimism, and from 6 to 12 high optimism (Reche et al., 2018b).

To measure resilience, the Resilience Scale (ER) designed by Wagnild and Young in 1987 (Wagnild and Young, 1993), and adapted to Spanish by Ruiz et al. (2012), was administered. This scale evaluates the degree of individual resilience and resilience as a positive personality characteristic, which favors adaptation. It is made up of 25 items, written in a positive way, assessed using a Likert-type response format, from 1 (strongly disagree) to 7 (strongly agree). Through this scale, the overall resilience score is obtained (the higher the score, the more resilience) and of these factors: personal competence (indicating self-confidence, independence, decisiveness, invincibility, power, resourcefulness, and perseverance) and acceptance of oneself and life (representing adaptability, balance, flexibility) (Reche and Ortín, 2013) and in Uruguayan judokas (Reche et al., 2014), finding an overall internal consistency of.89 in both studies (Reche et al., 2020).

Finally, the Inventory of Bumout in Athletes Revised (IBD-R; Garcés de Los Fayos Ruiz et al., 2012) was administered, with 19 items to measure the three dimensions of bumout proposed by Maslach and Jackson (1981): Emotional Exhaustion, Reduced Personal Accomplishment and Depersonalization, and with an overall internal consistency of 0.75. The response format was Likert-type, with five response alternatives. The items corresponding to Emotional Exhaustion and *Depersonalization* are formulated in such a way that the higher the numerical response of the subject, the greater the *burnout* experienced; while the items of *Reduced Personal Accomplishment* are formulated in the opposite direction: the lower the numerical response of the subject, the greater the degree of *burnout* experienced. In addition, there is an intermediate zone between P33 and P66 which, although it does not determine an exact level of *burnout*, marks a tendency or predisposition to suffer burnout in the future and represents moderate *burnout* (Tutte, 2009; Tutte et al., 2010).

### Procedure

Authorization was requested from the *Fundación Deporte Uruguay*, to carry out our research work with all the athletes selected and with the possibility of qualifying for the Pan-American Games held in Lima, Peru. Once this authorization was obtained, we proceeded to contact all the sports federations to administer the questionnaires to the athletes who agreed to participate voluntarily.

A day was set for the application of the techniques and a collective administration was carried out in a gymnasium, with an approximate duration of 30 min, directed by the researcher and four collaborators who explained to the athletes the instructions to be answered and answered any doubts that might arise. Athletes who for some reason did not attend that day were scheduled for another time to answer the questionnaires.

Each athlete was given a booklet in which the following information was requested: name, surname, age, years of experience in sports practice, type of sport, and hours of training. As well as the instruments. Emphasis was placed on the importance of data confidentiality and informed consent was requested beforehand. For those athletes who were under the age of majority, parents were asked to sign a consent form the days prior to the application of the questionnaires.

### Statistical analysis

Descriptive analyses (means, statistical deviation, skewness and kurtosis analysis, and normality tests) were performed for the continuous variable’s optimism and resilience, and for the dependent variable Burnout in its three dimensions: Emotional Exhaustion, Depersonalization, and Reduced Personal Accomplishment frequencies, percentages, and the corresponding Chi-square analysis, to finish with contingency tables to perform the crosses with the variables gender and type of sport. After performing the normality studies, we found that the variables did not have a normal distribution and proceeded to perform the non-parametric Mann–Whitney U test with Bonferroni correction, to analyze the presence of statistically significant differences between optimism and resilience with gender and type of sport. Finally, we performed a binary logistic regression, to explain the model that presents a dichotomous dependent variable Burnout (without burnout and with burnout symptomatology) in its three dimensions (Emotional Exhaustion, Depersonalization, and Reduced Personal Accomplishment) and the independent variables resilience and optimism.

Logistic regression analysis provides two types of information: the role played by each variable in the improvement of the regression model, and that relating to each variable included in the equation. This second type of information is usually provided in studies using this type of analysis, since it provides a regression coefficient of the independent variables (coefficient B, or odds ratio), whose significance allows us to interpret whether the variable increases the probability of having a certain value in the dependent variable which, in our case, is suffering from *burnout* symptoms.

The SPSS statistical package, version 28, was used for all analyses.

## Results

From the results found, we observe that 66.9% of the total sample presents Burnout symptomatology for the Emotional Exhaustion dimension, being 31.40% of women and 35.54% men. Meanwhile, considering the type of sport, 14% of the individual athletes presented symptoms and 26.4% of the team athletes. Regarding the depersonalization dimension, 52.1% of the sample showed Burnout symptoms, 19.86% being female, and 32.23% male. In turn, 23% of the individual athletes showed symptoms and 17.35% of the team athletes. Finally, in the dimension Reduced Personal Accomplishment, 17.4% of the sample had symptoms of Burnout, 5.78% being female, and 11.57% male. Continuing with the type of sport, 30.57% of individual athletes have symptoms and 9.92% of athletes who practice team sports. Table 2 shows the main results described.

**Table 2:** Percentages, frequencies, and Chi-square analysis of burnout according to gender and type of sport.

	Emotional exhaustion		Despersonalization		RRA	
	Woman	Man	Woman	Men	Woman	Men
Without *Burnout*	15 (12.4%)	25 (20.66%)	29 (23.96%)	29 (23.96%)	46 (38.01%)	54 (44.63%)
With symptoms of *Burnout*	38 (31.40%)	43 (35.54%)	24 (19.86%)	39 (32.23%)	7 (5.78%)	14 (11.57%)
	Individual	Collective	Individual	Collective	Individual	collective
Without *Burnout*	23 (19%)	49 (40.49%)	30 (24.79%)	42 (34.71%)	63 (52.06%)	9 (7.44%)
With symptoms of *Burnout*	17 (14.04%)	32 (26.44%)	28 (23.14%)	21 (17.35%)	37 (30.57%)	12 (9.92%)

The Chi-square (*X*^2^) analyses performed indicate that there are no statistically significant differences between the Burnout dimensions (Emotional Exhaustion, Depersonalization, and Reduced Personal Accomplishment) and gender (AE: *X*^2^ = .964a; *p* = 0.339; Desp: *X*^2^ = 1.739a; *p* = 0.204; RRP: *X*^2^ = 1.131a; *p* = 0.339). The relationship between Burnout levels and the type of sport (individual and collective) was similar, with no statistically significant differences (AE: *X*^2^ = 0.100; *p* = 0.844; Desp: *X*^2^ = 2.798a; *p* = 0.100; RRP: *X*^2^ = 2.922a; *p* = 0.141).

Table 3 allows us to observe the results obtained from the descriptive analyses, indicating a mean value of 134 on the Resilience scale, which would indicate that these athletes have a moderate level of resilience (range of 121–146, according to cut-off points taken in other studies). Likewise, it can be observed that in relation to the optimism variable, a moderate optimism profile is presented in the total sample (*M* = 5.2; SD = 3.71) taking as indicators a range of 3–5 points (Lacárcel et al., 2022).

**Table 3:** Descriptive analyses for the continuous variable’s resilience and optimism.

Scale	Variable	Half	Deviation (Dev.)	Asymmetry	Error estándar de asimetría	Kurtosis	Standard error of skewness	Kolmogorov–Smirnov	*p*
	
Resilience Scale	Personal competence	91.717	11.462	−1.483	0.221	5.003	0.438	10.95	0.000
	Acceptance of oneself and of life	42.322	5.611	–0.516	0.220	0.885	0.437	11.00	0.000
	Resilience Total	134.017	15.672	−1.446	0.221	4.989	0.438	10.954	0.000
	Optimism Total	5.20	3.71370	–0.933	0.221	2.709	0.438	0.095	0.001
LOT-R	Optimism	9.3333	1.94173	–1.127	0.221	3.18	0.438	10.863	0.000
	Pessimism	4.1333	2.55011	0.331	0.221	−0.235	0.438	8.879	0.000

When performing the normality analysis, through the Kolmogorov–Smirnov test, we found that the dimensions of the Resilience scale and those of the optimism variable do not have a normal distribution. To identify the presence of statistically significant differences between these variables and gender and type of sport, we used the non-parametric Mann–Whitney *U* test (*p* < 0.05), with the Bonferroni correction (*p* = 0.008). From this test, we found that there are statistically significant differences between the Personal Competence dimension and the type of sport (*p* = 0.007), with individual athletes presenting a higher mean (*M* = 67.59) in relation to collective athletes (*M* = 50.22). Although the variables optimism and total resilience present statistically significant differences between them and the type of sport, they do not comply with the correction performed by Bonferroni (optimism: *p* = 0.049; total resilience: *p* = 0.016). In relation to the dimensions of resilience and optimism and gender, the presence of statistically significant differences is not found.

Finally, Table 4 presents the binary logistic regression analyses that allow us to explain the role played by each variable in the improvement of the regression model, and the relative importance of each variable included in the equation.

**Table 4:** Coefficients and significance of the variable’s optimism and in the regression model for the dependent variable emotional exhaustion.

Subscales	B	E.E	Wald	Gl	Sig	Exp (B)
Personal Competence	0.037	0.027	1.883	1	0.170	1.038
Acceptance of oneself and of life	–0.188	0.057	10.903	1	<0.001	0.828
Optimism	–0.039	0.139	0.078	1	0.780	0.962
Pessimism	0.383	2.289	3.345	1	<0.001	1.467

Analyzing the odds ratio of the variable Acceptance of Life and oneself we find that the coefficient B is negative (Exp B = 0.828), when this occurs the odds ratio is always less than 1, showing that when there is an increase of one unit in the independent variable there is a decrease in the advantage of the event studied (Pardo, 2008). This would indicate that, for each extra point on the scale, the advantage of having some Burnout symptom decreases, 828 times, which means that each additional point on this subscale decreases the advantage of having some Burnout symptom by 17.2%. While observing the values obtained in the pessimism variable, the coefficient B associated with the variable is positive and significant (*p* < 0.001) with an advantage ratio Exp (B) = 1.467. It can be concluded that for each additional point on the pessimism scale, the advantage of having some Burnout symptom increases by 46.7%.

The rest of the variables studied do not present significant values, since they obtain a *p* > 0.05 for the dependent variable Emotional Exhaustion. The same occurs when analyzing the independent variables with the dependent variables Depersonalization and Reduced Personal Accomplishment, yielding values greater than statistical significance.

## Discussion and conclusions

The objective of this research was focused on describing and checking whether resilience and optimism as determinant psychological variables can be seen as preventive strategies against the appearance of burnout in sport. Variables that are related to important aspects such as the personality of the athlete, as well as their general style of functioning and the incidence of social variables, would affect the possibility of these to generate psychological skills of care and protection or, on the contrary, not to have them and be more vulnerable to the presence of this syndrome.

On the other hand, we had to observe the presence of burnout or any of its components in a sample of elite athletes, which represents 85% of the population we are addressing, selected Uruguayans to compete in the Pan-American Games, observing that the analysis of Emotional Exhaustion, Depersonalization, and Reduced Personal Accomplishment, leads to verify the existence of burnout in more than two-thirds of the sample (66.9%) present Burnout symptoms for the Emotional Exhaustion dimension, with the problematic casuistry that this implies, being the presence more evident in men and in team sports, although there are no significant differences attributable to gender or type of sport, being the above trends that will require future and further analyses. Although the diagnosis of the syndrome requires that an athlete presents symptomatology in three dimensions of burnout, these elevated results in at least one of them increase the vulnerability to suffer from the syndrome in the future. (Vives and Garcés de los Fayos, 2004; Tutte et al., 2010; Reche et al., 2014; Tutte and Reche, 2016; Isorna et al., 2019). This shows the importance of working on protective variables that allow the development of effective emotional tools, thus minimizing the chances of developing this disease (Trujillo-Torrealva and Reyes-Bossio, 2019).

One of the variables to be explored in depth is resilience, and in this study, we found a moderate level in the total sample, results that are like those found in other studies (Reche and Ortín 2013; Reche et al., 2014, 2020; Tutte and Reche, 2016; Reche et al., 2018b; Serrano-Nortes et al., 2021; Lacárcel et al., 2022). These data would suggest a coping capacity in these athletes that would enable them to cope better with the difficult moments they encounter in their sporting interaction, which, in turn, would allow them to make adaptive use of this capacity in most situations. In relation to gender, no statistically significant differences were found, as occurs in other studies (Tutte, 2015); however, males scored slightly higher in relation to total resilience capacity.

On the other hand, optimism is presented at moderate levels for this sample of Pan-American athletes, showing medium-high levels of optimism. This would indicate that the athletes would have adequate psychological resources to face adversity in most cases. Coincident results were found with high-performance athletes from various disciplines (Reche et al., 2014; Tutte and Reche, 2016; Reche et al., 2018a; Angosto et al., 2021), which is characteristic of athletes who maintain a performance associated with success. Specifically, it is worth highlighting the differences that we have found for the personal competence dimension and the type of sport, with individual athletes having the highest mean. Similar results have been found in studies that suggest the existence of variations depending on the sport practiced, indicating that individual athletes tend to have higher levels of resilience (Morgan et al., 2013; López-Suárez, 2014).

Although there are statistically significant differences between optimism and resilience and the type of sport, it does not meet the Bonferroni correction. It is always the individual sportsmen and women who present a higher mean. This has occurred in other studies where the results indicate that athletes who practice team sports tend to have lower levels of optimism (Laborde et al., 2016). Similarly, there are studies that make reference to the fact that optimism is a distinctive variable of athletes who practice competitive sports, as in this case, since it is a sample of high-performance athletes who participate in a meeting of great importance such as the Pan-American Games (Gould et al., 2002; López-Gullón et al., 2017).

As in our study, there are numerous investigations that associate resilience and optimism and emphasize the importance of these constructs in the sports context (Martin-Krumm et al., 2003; Reche et al., 2014; Tutte and Reche, 2016; Lacárcel et al., 2022), noting that the most optimistic athletes are, in turn, the most resilient ones (Souri and Hasanirad, 2011; Freche, 2013). Other studies point to a positive correlation between both constructs (Aranzana et al., 2016; Reche et al., 2018b), all impacting on an improvement in the psychological resources shown by such athletes to cope with specific problems, as is the case with burnout syndrome.

Finally, we would like to highlight that in relation to the regression analyses performed, we found statistically significant differences between the dependent variable Emotional Exhaustion and acceptance of life and self and pessimism. About the dimension of resilience, acceptance of life, and self, the results indicate that when this is present with high scores, the probability of developing burnout syndrome decreases significantly. Highlighting the importance of working on this variable that allows a positive adaptation to situations of adversity (Aranzana et al., 2021), where athletes with high levels could face more effectively the challenges and difficulties that the sports context may bring them. Other research has found that athletes with burnout symptomatology have a less resilient and optimistic profile (Tutte, 2015; Tutte and Reche, 2016).

As for pessimism, although the results indicate that pessimism has a positive relationship with the possibility of suffering burnout, and when it is present in high levels, it significantly increases the probability of the syndrome appearing, other studies reach similar conclusions through the analysis of optimism. Some research found that more optimistic individuals had less reduction in mental vigor, suggesting the benefit of optimism in coping with potentially stressful situations (Brydon et al., 2009). At the same time, optimism had a significant negative relationship with both stress and burnout (Gustafsson and Skoog, 2012; Gustafsson et al., 2018; Angosto et al., 2021). It does not matter, therefore, whether we focus on optimism or pessimism, but on the relationship, it has with the possibility of developing burnout. We can generate strategies aimed at optimizing existing positive approaches to protect athletes from the onset of this syndrome or, on the contrary, develop interventions that aim to make pessimism more flexible and teach tools to improve emotional management and coping strategies to minimize or delay the effects of burnout (Reyes-Bossio et al., 2012; Lundgren et al., 2020). It is therefore essential to design interventions that teach emotional skills to manage adversity and prevent burnout (Sallen et al., 2018; Guerrero and Magalhaes, 2019; Cárcamo-La Torre and Reyes-Bossio, 2022).

We conclude by stating that the present work provides some lines of future research and application that will have to be addressed to eliminate the limitations that the study, by its very configuration, has shown.

Firstly, it seems obvious that resilience and optimism as burnout preventive psychological skills should also be considered as potential prevention strategies if they are introduced as skills to be improved in psychological training with elite athletes. Secondly, it would be interesting to focus both the research and the practical application of the work of the sport psychologist in the sense of orienting the help toward team sports athletes, with greater intensity, since it is demonstrated in the work that they present higher levels of burnout, at the same time as worse results in terms of resilience and optimism, in a general way.

Thirdly, it seems that the results guide us in the sense of understanding emotional exhaustion as the essential variable in terms of the appearance of the syndrome, which, as our results show, will be closely related to the appearance and potential relationships with the personal competence of the athlete, as well as his or her personal profile of pessimism versus optimism, proving to be an essential component in the future study of burnout in elite athletes.

Fourth, the data provided allow us to glimpse some evidence that should be addressed in future research, such as the role that personality characteristics could play in the learning, existence, and maintenance of skills related to resilience and optimism. Finally, it is interesting to continue to study burnout and its relationship with these variables because it is perhaps the central axis on which to understand other associated variables, in terms of their presence or not in athletes affected by the syndrome, such as motivation or self-confidence, to cite just two examples.

## Data availability statement

The raw data supporting the conclusions of this article will be made available by the authors, without undue reservation.

## Ethics statement

The studies involving human participants were reviewed and approved by the Ethics Council of the Faculty of Psychology of the Catholic University of Uruguay. We have the support and approval of the Fundación Deporte Uruguay, who provided a letter of support to apply for a competitive research fund. The participants provided their written informed consent to participate in this study.

## Author contributions

All authors listed have made a substantial, direct, and intellectual contribution to the work and approved it for publication.

## Conflict of interest

The authors declare that the research was conducted in the absence of any commercial or financial relationships that could be construed as a potential conflict of interest.

## Publisher’s note

All claims expressed in this article are solely those of the authors and do not necessarily represent those of their affiliated organizations, or those of the publisher, the editors and the reviewers. Any product that may be evaluated in this article, or claim that may be made by its manufacturer, is not guaranteed or endorsed by the publisher.
